# Management of acute metabolic acidosis in the ICU: sodium bicarbonate and renal replacement therapy

**DOI:** 10.1186/s13054-021-03677-4

**Published:** 2021-08-31

**Authors:** Kosuke Yagi, Tomoko Fujii

**Affiliations:** 1 Intensive Care Unit, Jikei University Hospital, Tokyo, Japan; 2 ANZIC-RC, Monash University School of Public Health and Preventive Medicine, Melbourne, VIC Australia

## Abstract

This article is one of ten reviews selected from the Annual Update in Intensive Care and Emergency Medicine 2021. Other selected articles can be found online at https://www.biomedcentral.com/collections/annualupdate2021. Further information about the Annual Update in Intensive Care and Emergency Medicine is available from https://link.springer.com/bookseries/8901.

## Introduction

Metabolic acidosis is a process caused by an increase in weak acids or a decrease in strong ion difference (SID) [1]. Serum proteins, albumin, and inorganic phosphate are considered as weak acids. Strong ions, such as Na^+^, K^+^, Ca^2+^, Mg^2+^, and Cl^−^, exist at a fully ionized status in body fluids. SID is the presence of an excess of strong cations over strong anions, and the normal value in plasma is 42 mEq/l. The method to quantify metabolic acidosis using SID and weak acids was introduced by Stewart in the 1980s and still creates debate in its clinical application [2]. Plasma base excess is widely used to identify a metabolic component of acidosis in clinical practice. The base excess approach was shown to be equivalent to Stewart’s SID approach in quantifying acid–base status in critically ill patients [3].

Metabolic acidosis is classified into acute and chronic. Although it is not clearly defined, acute metabolic acidosis occurs within a few days. Chronic acidosis is a condition that lasts for weeks or even years [4]. In this chapter, we focus on acute metabolic acidosis in intensive care unit (ICU) patients and provide an update from recently published clinical studies.

## Epidemiology of metabolic acidosis in the ICU

Acute metabolic acidosis is well-recognized in the ICU. However, epidemiological data are scarce, which has limited our understanding of the approach to metabolic acidosis until recently.

A retrospective observational study using a large bi-national ICU database in Australia and New Zealand examined the incidence, characteristics, and outcomes of patients with various definitions of metabolic acidosis [5]. Severe metabolic acidosis was defined as a pH ≤ 7.20, PaCO2 ≤ 45 mmHg, HCO3^−^  ≤ 20 mmol/l, and total sequential organ failure assessment (SOFA) score ≥ 4 or lactate ≥ 2 mmol/l [6] and occurred in 1.5% of the patients in the ICU. The ICU and hospital mortality rates of these patients were 43.5% and 48.3%, respectively. Moderate or severe metabolic acidosis was defined as pH < 7.30, base excess < −4 mmol/l and PaCO2 ≤ 45 mmHg, and occurred in 8.4% of ICU patients. The ICU and hospital mortality rates were 17.3% and 21.5%, respectively [5]. The mortality of patients with moderate or severe metabolic acidosis was higher than that of patients with sepsis observed in the same database [7], suggesting the clinical relevance of improving care for patients with metabolic acidosis.

A French multicenter prospective study described the incidence of severe acidemia in five ICUs [8]. Severe acidemia was defined as pH < 7.2, including respiratory acidosis, metabolic acidosis, and mixed acidosis. This severe acidosis occurred in 8% (200/2550) of the patients within 24 h of ICU admission. After excluding patients with diabetic ketoacidosis (DKA), which is adjudicated to be an entity with a low risk of death, and patients with respiratory acidosis, ICU mortality of patients with metabolic or mixed severe acidosis was as high as 57% (89/155) [8].

A recently published international observational study conducted in 18 ICUs in Australia, Japan, and Taiwan reported that 14% (1292/9437) of critically ill patients had moderate or severe metabolic acidosis [9]. The median incidence of metabolic acidosis at a study ICU was 172.5 patients/year, suggesting that the management of metabolic acidosis is a relevant issue in patient care in the ICU.

## Common types of metabolic acidosis in the ICU

The causes of acute metabolic acidosis are diverse in critically ill patients. DKA, lactic acidosis, and hyperchloremic acidosis are responsible for most cases of severe metabolic acidosis cases due to a decreased SID [10].

DKA is a medical emergency in patients with diabetes mellitus as a result of insulin deficiency. The hepatic metabolism of fatty acids produces beta- hydroxybutyrate and acetoacetate, strong anions in the human body. As hyperglycemia induces osmotic diuresis, patients with DKA have a markedly reduced extracellular fluid volume. The available evidence was summarized in a review that revealed the paucity of sufficient data on clinical impact [11]. To date, a comprehensive epidemiological study on DKA investigating its epidemiology and clinical outcomes is still lacking.

Lactate is a strong anion in the human body as more than 99% of lactate is ionized. Lactic acidosis is observed in cardiogenic or hypovolemic shock, severe heart failure, severe trauma, and sepsis [8], with high mortality rates, ranging from 30 to 88% depending on the definition used [12, 13]. The mortality of patients with lactic acidosis was reportedly the highest (56%) amongst patients with metabolic acidosis defined by a SBE (standard base excess) < -2 mEq/l [14].

Recently, hyperchloremic acidosis caused by intravenous fluid products has become widely known and is reported in 19% to 45% of patients in the ICU [14, 15]. Table 1 shows the electrolytes and SIDs of intravenous fluid products commonly used in the ICU. Theoretically, acidosis occurs when intravenous fluid products with a SID lower than that of the patient’s plasma are administered. Balanced crystalloids, i.e., Ringer’s acetate, Ringer’s lactate, and Plasmalyte, contain acetate, lactate, or gluconate to replace chloride. Those strong anions do not contribute to SID as they are metabolized by the liver faster than renal chloride excretion.

**Table 1:** Electrolytes and strong ion difference (SID) of fluid products commonly used in the intensive care unit (ICU)

Ion	Concentration (mEq/L)						
	Plasma	0.9% NaCl	Ringer’s lactate	Ringer’s acetate	5% albumin	Plasma-Lyte 148	8.4% NaHCO3
**Na**^**+**^	140	154	131	130	148	140	1000
**K**^**+**^	5	0	5	4	0	5	0
**Cl**^**−**^	100	154	111	109	131	98	0
**Ca**^**2+**^	2.2	0	2	3	0	0	0
**Mg**^**2+**^	1	0	1	0	0	1.5	0
HCO3^−^	24	0	0	0	0	0	1000
Lactate^−^	1	0	29	0	0	0	0
Acetate^−^	0	0	0	28	0	27	0
Gluconate	0	0	0	0	0	23	0
SID	47.2	0	28	28	17	48.5	1000

Ions in bold font contribute to SID

## Why metabolic acidosis matters

Metabolic acidosis can have various adverse effects, but the most critical consequence is its effect on the cardiovascular system. Recognition of this effect dates back to the 1960s when a study reported reduced cardiac contractility at pH < 7.1 when lactic acid was administered to dogs [16]. Animal experiments were also performed on dogs given lactic acid and hydrochloric acid to produce lactic acidosis and hyperchloremic acidosis. The dogs were given epinephrine, norepinephrine, and dobutamine to counteract the shock status. The cardiac index decreased when epinephrine or norepinephrine was administered; however, dobutamine administration increased the cardiac index. This result suggested that acidosis decreased the catecholamine reactivity to norepinephrine or epinephrine [17]. Pedoto et al. reported that when hydrochloric acid was administered to rats to mimic hyperchloremic acidosis, nitric oxide (NO) production increased, provoking vasodilation, and resulting in reduced systemic blood pressure [18]. Fatal arrhythmias induced by acidosis have also been reported in an experimental model [19]. However, clinical studies in humans have not yet demonstrated a causal relationship between metabolic acidosis and cardiovascular dysfunction [20–22].

## How we manage metabolic acidosis in the ICU

Metabolic acidosis in critically ill patients is not a single disease but a syndrome driven by various underlying conditions. As such, the basic principle is to treat the underlying cause of metabolic acidosis. Sodium bicarbonate may be administered if there is a concern for the suppressed cardiac function that metabolic acidosis may cause. The rationale for using sodium bicarbonate for metabolic acidosis is that the intravenous administration of a high SID solution would increase the pH, resulting in improved cardiac function.

The evidence on the biochemical effects of intravenous sodium bicarbonate in acute metabolic acidosis has been systematically reviewed [23]. The summary of 12 relevant studies showed that pH, serum bicarbonate, base excess, serum sodium, and PaCO2 increased during and after the intravenous administration of sodium bicarbonate [23]. By contrast, serum anion gap and potassium decreased. Some concern was raised about intracellular acidosis due to the back-diffusion of CO2 and decreased ionized calcium that might impair cardiac contraction. However, there was no consistent evidence from the literature review that sodium bicarbonate administration was associated with decreased ionized calcium or decreased cardiac output [20, 24].

The effects of sodium bicarbonate on clinically relevant outcomes should be investigated in RCTs. The systematic review [23] identified only two RCTs that have been conducted [5, 25]. Hoste et al. compared the effect of sodium bicarbonate and tris(hydroxymethyl)aminomethane, THAM, in 18 patients with mild metabolic acidosis [25]. The trial, published in 2005, did not report clinically important out-comes, perhaps because the trial was conducted as a pilot trial. THAM has not been explored for its effects since this trial and is rarely used in current clinical practice.

An important RCT investigating the effects of sodium bicarbonate for severe metabolic acidosis was published in 2018 [5]. The BICAR-ICU trial was conducted in 26 French ICUs and enrolled 389 patients with severe acidemia (pH ≤ 7.20, PaCO2 ≤ 45 mmHg, HCO3^−^  ≤ 20 mmol/l, and total SOFA score ≥ 4 or lactate ≥ 2 mmol/l). The trial excluded patients with DKA or chronic kidney disease (CKD). Patients were allocated to an intervention group receiving 4.2% sodium bicarbonate to maintain pH > 7.3 throughout the ICU stay or a control group with usual care. There was no difference in the primary outcome, which was a composite of death by day 28 and at least one organ failure at day 7, between the groups. However, treatment with sodium bicarbonate was associated with a reduced need for renal replacement therapy (RRT) in the ICU. Furthermore, in the pre-specified subgroup of patients with acute kidney injury (AKI) (AKIN score 2 or 3), sodium bicarbonate was associated with improved survival and reduced need for RRT [5].

In a retrospective, observational study using the Medical Information Mart for Intensive Care (MIMIC)-III database, sodium bicarbonate administration was not associated with improved survival in patients with metabolic acidosis (pH < 7.3, HCO3^−^  < 20 mmol/l and PaCO2 < 50 mmHg) but was associated with improved survival in septic patients with stage 2 or 3 AKI and severe acidemia (pH < 7.2) [26].

A recent international observational study revealed that 18% of patients with moderate or severe metabolic acidosis receive sodium bicarbonate in current clinical practice [9]. However, the total amount of sodium bicarbonate given during the first 24 h of metabolic acidosis was 110 mmol, which was not adjusted for body weight or base excess. The study also reported that sodium bicarbonate administration was possibly associated with lower ICU mortality in acidotic patients with vasopressor dependency, albeit with a lack of statistical significance. Given that the rationale to use sodium bicarbonate would be to support cardiovascular function, this finding provides a sound basis for further investigation on the effect of sodium bicarbonate in patients with metabolic acidosis and on vasopressors.

## Sodium bicarbonate for subtypes of metabolic acidosis

Administration of sodium bicarbonate has been considered for DKA not only because sodium bicarbonate reverses the acidotic status but because acidosis possibly contributes to insulin resistance [27]. However, a retrospective single center study from the USA reported that sodium bicarbonate administration in the emergency department was not associated with time to resolution of acidosis in patients with DKA with a pH < 7.0 [28]. There was also no difference in hospital length of stay [28]. A systematic review in 2011 found that sodium bicarbonate did not shorten the duration of acidosis, ketosis, or glycemic levels [11]. Furthermore, there was a high incidence of hypokalemia that required correction in patients who received sodium bicarbonate [11]. These findings imply that the beneficial effects of sodium bicarbonate administration for DKA might be limited. However, the systematic review by Chua et al. revealed a lack of rigorous randomized clinical trials that assessed patient-centered outcomes in these patients [11].

Sodium bicarbonate for lactic acidosis has been compared with saline in two small-scale randomized, crossover, single center trials [20, 21]. Cooper et al. reported that sodium bicarbonate administration increased pH and PCO2 with no change in blood pressure or cardiac output [20]. Similarly, Mathieu et al. found an increase in pH but no change in hemodynamic parameters, including cardiac index [21].

For cardiac arrest, several observational studies have reported an increase in the rate of return of spontaneous circulation in patients receiving sodium bicarbonate [29–32]. However, one study found that this treatment was associated with a worse survival rate and neurological outcomes to hospital discharge [33]. A pilot RCT showed no improvement in patient mortality [34], return of spontaneous circulation rate, or neurologically favorable status in treated patients [35]. At present, routine use of sodium bicarbonate is not recommended for cardiopulmonary resuscitation [36].

## Renal replacement therapy for metabolic acidosis

There has been no clear consensus of clinical indications for RRT; however, severe acidosis is a commonly accepted indication. In RCTs on the timing of RRT that have been published over the past 5 years, i.e., the AKIKI trial, the IDEAL-ICU trial, and the STARRT-AKI trial, metabolic acidosis with severe acidemia was used as one of the absolute indications [37–39].

The AKIKI trial was a multicenter RCT in France, enrolling patients with stage 3 AKI, in which 67% of the patients had septic shock [37]. The trial compared early initiation of RRT in stage 3 AKI and delayed initiation with absolute indications. The absolute indications for RRT included severe acidemia with pH < 7.15, either metabolic acidosis or mixed acidosis. Of note, 21% of the trial participants in the control group received RRT for metabolic acidosis [37].

The IDEAL-ICU trial was another multicenter RCT conducted in France, enrolling patients with septic shock and stage 3 AKI [38]. The absolute indications for RRT included metabolic acidosis with pH < 7.15 and base deficit > 5 mEq/l or HCO3^−^  < 18 mEq/l. Among the patients who received RRT for the absolute indication, 13.4% met the metabolic acidosis criteria [38].

The STARRT-AKI trial was the largest international RCT, including 3019 patients from 15 countries [39]. The main aim of the trial was to assess whether an accelerated strategy to start RRT at stage 2 or 3 AKI would improve patient-centered outcomes compared with a delayed initiation with absolute indications. The absolute indications for RRT included severe acidemia and metabolic acidosis, defined as pH ≤ 7.2 or HCO3^−^  < 12 mmol/l. Of the patients treated with RRT, 16.6% met the criteria for severe metabolic acidosis [39].

From the STARRT-AKI trial and the BICAR-ICU trial, patients with stage 2 or 3 AKI should avoid immediate intervention with RRT and may benefit from sodium bicarbonate if severe metabolic acidosis is present despite appropriate treatment for underlying conditions.

## Agenda for future research

Recent clinical research, including large RCTs, has provided new evidence and advanced our understanding of the management of metabolic acidosis. However, high-quality data from rigorous clinical research to guide standard practice are still lacking. Research priorities include the following:

- The benefits and harms of sodium bicarbonate on cardiovascular function
- Sodium bicarbonate not only for severe metabolic acidosis but for moderate metabolic acidosis
- Sodium bicarbonate for severe metabolic acidosis with stage 2 or 3 AKI (BICARICU-2, Clinicaltrials.gov identifier NCT04010630, in progress).

## Conclusion

We have reviewed the recent clinical data on epidemiology and management of metabolic acidosis. Metabolic acidosis is common in the ICU, and even moderate metabolic acidosis carries higher mortality than severe sepsis. Sodium bicarbonate or RRT is used occasionally to normalize acid–base imbalance due to metabolic acidosis in the ICU; however, high-quality evidence is still limited. Patients with severe metabolic acidosis and stage 2 or 3 AKI might be a possible target population for sodium bicarbonate administration. Further clinical trials are required to provide more robust information in a clinically relevant patient population.

## Data Availability

Not applicable.
